# Optimal guideRNAs for re-directing deaminase activity of hADAR1 and hADAR2 in *trans*

**DOI:** 10.1093/nar/gku272

**Published:** 2014-04-15

**Authors:** Marius F. Schneider, Jacqueline Wettengel, Patrick C. Hoffmann, Thorsten Stafforst

**Affiliations:** Interfaculty Institute of Biochemistry, University of Tübingen, Tübingen 72076, Germany

## Abstract

Adenosine deaminases that act on RNA (ADAR) are a class of enzymes that catalyze the conversion of adenosine to inosine in RNA. Since inosine is read as guanosine ADAR activity formally introduces A-to-G point mutations. Re-addressing ADAR activity toward new targets in an RNA-dependent manner is a highly rational, programmable approach for the manipulation of RNA and protein function. However, the strategy encounters limitations with respect to sequence and codon contexts. Selectivity is difficult to achieve in adenosine-rich sequences and some codons, like 5′-GAG, seem virtually inert. To overcome such restrictions, we systematically studied the possibilities of activating difficult codons by optimizing the guideRNA that is applied in *trans*. We find that all 5′-XAG codons with X = U, A, C, G are editable *in vitro* to a substantial amount of at least 50% once the guideRNA/mRNA duplex is optimized. Notably, some codons, including CAG and GAG, accept or even require the presence of 5′-mismatched neighboring base pairs. This was unexpected from the reported analysis of global editing preferences on large double-stranded RNA substrates. Furthermore, we report the usage of guanosine mismatching as a means to suppress unwanted off-site editing in proximity to targeted adenosine bases. Together, our findings are very important to achieve selective and efficient editing in difficult codon and sequence contexts.

## INTRODUCTION

**Figure 1.**
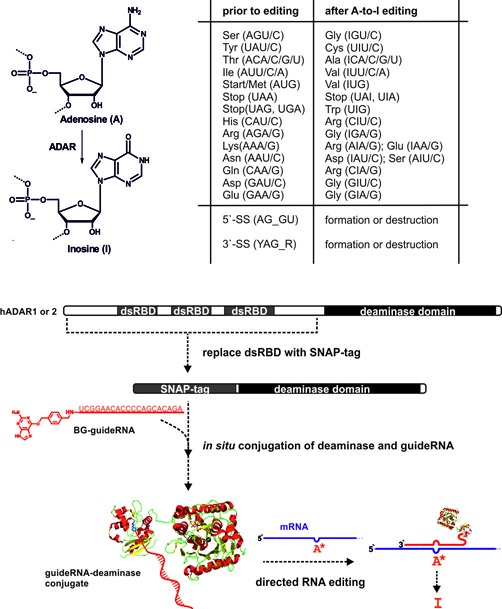
Hydrolysis of the exocyclic amino group of adenosine by ADAR enzymes results in formation of inosine that is biochemically read as guanosine. Twelve out of the 20 canonical amino acids are potentially targetable with A-to-I editing. The scope also includes various RNA processing elements as splice sites (SS) for instance. However, some codons represent difficult substrates usually not accepted by ADAR enzymes. To direct ADAR activity toward new, user-defined targets, the dsRNA binding domains (dsRBD) have been replaced by a SNAP-tag (an engineered O6-alkylguanine-DNA-alkyl transferase). The latter allows for the covalent conjugation of the SNAP-ADAR fusion with benzylguanine (BG)-modified guideRNAs that direct the enzyme toward new targets. Fine-tuning of the guideRNA/mRNA duplex affords control over editing efficiency and selectivity.

Adenosine deaminases acting on RNA (ADAR) promote hydrolysis of adenosine to inosine in double-stranded RNA (dsRNA) substrates; see Figure 1 (1,2). Since inosine is read as guanosine, A-to-I editing can have profound effects on the RNA transcript. Editing in the open reading frame (ORF) can lead to the substitution of single amino acids. Editing in the introns or untranslated regions can change the processing and regulation of the transcript. Knocking down ADAR enzymes in mammals gives severe phenotypes and demonstrates their essential role for the functioning of the nervous and immune system (1,2). Aberrant editing is associated with mental disorders (3). Furthermore, editing interferes with virus propagation and RNA interference (4–6).

Beyond its endogenous cellular role, A-to-I editing represents an attractive enzymatic activity for reprogramming genetic information on the RNA level. Out of the 20 canonical amino acids, 12 are potentially editable, including most of the residues that are essential for enzyme catalysis, protein signaling or protein glycosylation for instance; see Figure 1. Thus, editing can have a strong impact on protein function. Beside this, functional elements like STOP and START codons, splice sites, splice modulating elements or polyadenylation sites are A- and G-rich and hence can also potentially be manipulated (7). Thus, we got interested in re-directing editing activity toward new, user-defined mRNAs. The natural enzymes find their dsRNA substrates via N-terminal RNA binding domains. Even though this recognition is structurally well understood (8), it does not seem feasible to reprogram the respective protein domains in a rational way. Instead, we re-engineered the protein-guided hADAR1 into an RNA-guided deaminase by covalently attaching the isolated deaminase domain of hADAR1 to a short guideRNA (9). Covalent attachment was achieved by fusion of a SNAP-tag to the N-terminus of the deaminase. Incubating such a fusion with 5′-*O*-BG-modified guideRNAs gives defined 1:1 conjugates in quantitative yields; see Figure 1. We demonstrated that such conjugates allow for the highly selective and efficient repair of nonsense and missense mutations in reporter genes (9). Furthermore, we could demonstrate that optimizing the positioning of the guideRNA allows for suppressing unwanted over-editing of proximate off-site adenosine residues to some extent (9). More recently we established our approach in cell culture and reported on the beneficial effects of Antagomir-like chemical modification of the guideRNA in a cellular environment (10). Chemical modification turned out as a means to fine-tune editing selectivity in a very delicate adenosine-rich sequence context as required for the repair of the F5 Leiden polymorphism (10). Others have recently reported a similar strategy to re-direct human ADAR activity in a guideRNA-dependent manner toward user-defined mRNAs and reported the repair of an UAG stop mutation in the CFTR gene in oocytes (11).

Today, the global editing of long dsRNA substrates by ADAR enzymes is well characterized (12). The enzymes prefer specific codon triplets around the targeted adenosines. The specificity is mainly determined by the deaminase domain itself and thus full-length proteins behave similar as the isolated deaminase domains. Most important for editing efficiency is the 5′-neighbor of the targeted adenosine. The preference is U > A > C >> G for hADAR1 and hADAR2 as well as for the isolated deaminase domains. Compared to the 5′-neighbor, the preference for the 3′-neighbor is much less distinct**;** however, a weak preference of G > U was found. Whereas the global editing specificity is clear, the understanding of highly specific, single editing events, as they happen in neuronal receptor genes like gluR for instance, is much more complex and requires the understanding of the contribution of the dsRNA binding domains (8).

**Figure 2.**
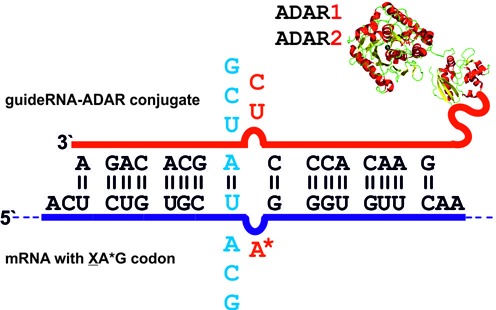
Editing set-up to study the influence of the 5′-neighbor: the XAG codon (X = U, A, C, G) in GFP mRNA (dark blue) was targeted by SNAP-ADAR/guideRNA conjugates in a way that the targeted adenosine (A*) was either base paired with U or mismatched with C, whereas the 5′-neighboring base X was either base paired or mismatched in all possible combinations. Both deaminase domains of hADAR1 and hADAR2 were studied.

To fulfil the full potential of directed RNA editing as a method for the rational manipulation of genetic information, we have to achieve highly specific editing of a single adenosine in neighborhood of other adenosines. We also need to find ways to activate editing of non-preferred codons, for instance, of glutamate (5′-GAR) and aspartate (5′-GAY). For this, we have now systematically studied the editing of four codons (UAG, AAG, CAG and GAG). Overall we looked at 64 editing settings that cover all possible combinations of matches and mismatches at the direct 5′-neighbor, that cover the usage of uridine or cytosine as the counter base, and that cover both, the deaminase domain of hADAR1 and hADAR2; see Figure 2. For the codons UAG, AAG and CAG, we report solutions that allow high editing yields (≥90%). We also found conditions under which the difficult GAG codon can be edited up to 50%, a number that would often be sufficient to attenuate disease phenotypes caused by loss-of-function mutations. Notably, we find clear differences for the deaminase domains of hADAR1 and hADAR2 and we apply unprecedented secondary structures to efficiently activate some of the difficult codons. In contrast to ADAR1 and ADAR2, no editing activity has ever been described for ADAR3 (13). ADAR3 is expressed highly tissue-specific only in the brain. Due to its lack of editing activity and close relationship to ADAR2, it was speculated that ADAR3 may modulate editing activity of ADAR1 and ADAR2 either by binding to the same substrates or by forming heterodimers with ADAR1 and ADAR2 (13). Since ADAR3 is in principle containing all elements essential for editing there remains the possibility of ADAR3 being active on an unknown substrate. Thus we also included SNAP-ADAR3 in our systematic study.

## MATERIALS AND METHODS

### SNAP-ADAR1, SNAP-ADAR2 and SNAP-ADAR3

SNAP-ADAR1 has been expressed, isolated and purified from YVH10 yeast as recently described (9). The gene of the deaminase domain of human adar2 (BC065545, sequenced clone from a commercial cDNA library) or human adar3 (BC140852) was subcloned via the AscI and BamHI restriction sites into the SNAP-ADAR1 construct replacing the ADAR1 deaminase domain of our initial construct (9,14) by using forward primer 5′-d(TAG GCG CGC CAG GGT CTG GCG GCG GCA GTA AGA AGC TTG CCA AGG CCC GG) and backward primer 5′-d(GCG GAT CCT ATT AAT GGT GAT GGT GAT GGT GGG GCG TGA GTG AGA ACT GGT C) for ADAR2, and forward primer 5′-d(TAG GCG CGC CAG GGT CTG GCG GCG GCA GTA AGA AGC TGG CCC GGG GTC AG) and backward primer 5′-d(GCG GAT CCT ATT AAT GGT GAT GGT GAT GGT GGA GAG TCA GTA GAA ACT GCT GCT G) for ADAR3. The plasmid is based on the yeast/*Escherichia coli* shuttle vector pRS426 (15). The fusion protein is under control of a Gal1-10 promotor, adding a C-terminal 6xHis-tag. To allow usage of BamHI, a natural BamHI site in the ADAR2 gene was disrupted by a silent point mutation using forward primer 5′-d(GGC ATC CAG GGT TCC CTG CTC AG) and the backward primer 5′-d(CTG AGC AGG GAA CCC TGG ATG CC) via overlap extension polymerase chain reaction (PCR). Compared to the human reference genome, the cDNA of ADAR3 contained a single SNP (single nucleotide polymorphism) in the deaminase domain (Ala625→Thr). To change this back into the reference sequence, a point mutation was introduced via overlap extension PCR with forward primer 5′-d(GTG AGT GAC GCC GAA GCG CGC CAG) and backward primer 5′-d(CTG GCG CGC TTC GGC GTC ACT CAC). Phusion polymerase (New England Biolabs) was used in all cloning steps. All PCR products were purified by 1.4% agarose gel electrophoresis. Ligation products were transformed into Xl1blue *E.* *coli*. After mini-preparation, plasmids were sequenced over the promotor and ORF.

All fusion proteins were produced on 1 L scale in YVH10 (16), similar to a literature protocol (17). For this the SNAP-ADAR1/2/3 genes in pRS426 were transformed into chemically competent YVH10 using the Frozen-EZ Yeast Transformation II Kit (Zymo Research). Cells were grown in SD-CAA media (20 g dextrose/L, 6.7 g/L yeast nitrogen base with ammonium sulfate, 5 g/L casamino acids, 100 mM sodium phosphate pH 6.0) supplemented with Trp (40 mg/L) and inositol (20 mg/L) for 4 days at 28°C, 200 revolutions per minute (rpm) in culture flasks and switched to SG-CAA (as SD-CAA but with dextrose being replaced by galactose) supplemented with Trp (40 mg/L), inositol (20 mg/L) and raffinose (10 g/L) for induction. After 4.5 days induction at 20°C, 200 rpm, cells were harvested, suspended with two volumes 10 mM imidazole, 750 mM NaCl, 20 mM Tris-HCl pH 8.0, 5% glycerol, lysed with a French press (20 000 Psi, 3 runs), and were clarified from the debris by centrifugation (40 000 g, 1 h). The lysates were subjected to a pre-equilibrated Ni-NTA gel via a 0.4 μm PES (poly ether sulfone) syringe filter. After loading, the column was washed with step-wise decreasing the salt content of the buffer, finally arriving at 10 mM imidazole, 100 mM NaCl, 20 mM Tris-HCl pH 8.0, 5% glycerol. The protein was eluted into 15 ml 400 mM imidazole, 100 mM NaCl, 20 mM Tris-HCl pH 8.0, 5% glycerol. The protein containing fractions were directly subjected to a 1 ml HiTrap Heparin column (GE-Healthcare) equilibrated with 100 mM NaCl, 20 mM Tris-HCl pH 8.0, 5% glycerol. The proteins were manually eluted by step-wise increasing salt concentration. Typically, the proteins were eluted between 250 and 350 mM NaCl and were >90% clean as judged from SDS–polyacrylamide gel electrophoresis (PAGE). The presence of the SNAP-tag was confirmed by staining the protein with *O*-BG-modified fluoresceine. Since yeast is not containing endogenous ADAR enzymes that could interfere with our construct, no further purification was done. The proteins were concentrated to a final volume of 250 μl with a 15 ml 10 kDa MWCO Amicon centrifugal filter and were changed to 150 mM NaCl without changing the other buffer conditions. The concentrations of the protein solutions were estimated by UV-spectroscopy with extinction coefficients of 600 cm^−1^ mM^−1^ (230 nm), 85 cm^−1^ mM^−1^ (260 nm) and 120 cm^−1^ mM^−1^ (280 nm) prior to addition of DTT (dithiothreitol). The protein stock solution was filled up with 86% glycerol to a total concentration of 20–25% glycerol and DTT was added to a final concentration of 2 mM. Proteins were aliquoted and stored at a concentration of ≥10 μM at −20°C. SNAP-ADAR1/2 loose dramatically in activity when they are diluted to less than 200 nM in the absence of bovine serum albumin. A typical 1 l production resulted in 15 nmol SNAP-ADAR1 (1 mg), 30 nmol SNAP-ADAR2 (2 mg), or 90 nmol SNAP-ADAR3 (6 mg).

### Reporter mRNA substrates

All mRNA substrates were transcribed with T7 RNA polymerase from the respective templates, as described before (9). mRNAs were treated with DNaseI and the disappearance of the template was controlled by PCR with Taq. mRNAs were purified by spin columns (Minelute Kit Qiagen). Templates containing the AAG, CAG and GAG codons at protein position 66 of the full-length egfp gene were obtained from the UAG containing Stop66 egfp template by overlap-extension PCR as described before (9). Primers for overlap-extension PCR were purchased from MWG Eurofins (Frankfurt) at cloning oligo purity. Codon AAG: forward primer 5′-d(CTA CTC TGT GCA AGG GTG TTC AAT GC) and backward primer 5′-d(GCA TTG AAC ACC CTT GCA CAG AGT AG); codon CAG: forward primer 5′-d(CTA CTC TGT GCC AGG GTG TTC AAT GC) and backward primer 5′-d(GCA TTG AAC ACC CTG GCA CAG AGT AG); GAG codon: forward primer 5′-d(CTA CTC TGT GCG AGG GTG TTC AAT GC) and backward primer 5′-d(GCA TTG AAC ACC CTC GCA CAG AGT AG). All DNA templates were sequenced prior to their usage in transcription. Template synthesis for Tyr65 and Ser67 missense GFP (green fluorescent protein) has been described before (9).

### guideRNAs

All guideRNAs were purchased as HPLC-cleaned, desalted 5′-C6-aminolinker-modified 20mer RNAs at 50 nmol scale from MWG Eurofins (Frankfurt). They were modified via the 5′-aminolinker with *O*-6-BG to allow later conjugation with the SNAP-tag. In a typical procedure, 150 μg NH_2_-gRNA (20–25 nmol) were dissolved in 25 μl Hepes-NaOH buffer (75 mM Hepes, 50 mM NaCl, pH 8.1) and were given to a pre-activated OSu-ester of BG-linker-COOH (10 equivalents BG-linker-COOH, 9 equivalents EDCI*HCl, 14 equivalents N-hydroxysuccinimide, 40 equivalents Hünig base, all in 25 μl DMSO (dimethylsulfoxide), 1 h pre-activation at 30°C; structure of BG-linker-COOH; see Supporting Information of reference (9)). After 1 h at 30°C, another 10 equivalents pre-activated BG-linker-OSu in 25 μl DMSO were added and incubated for 1 h at 30°C. The raw guideRNA was precipitated with ethanol-NaOAc, taken up in 1x TBE, 7 M urea and purified on a 20% 19:1 1x TBE-7 M urea PAGE mini gel (10 × 10 cm, 1 mm thick), cut out on a thin-layer chromatography plate under low-intensity 254 nm UV-light and was isolated by the crush-soak method into RNase-free water at 4°C overnight. To remove urea and buffer salts, the BG-guideRNAs were again precipitated, washed, dried and dissolved into 80 μl RNase-free water. Typically, 80–100% conversion was observed and around 40% pure BG-modified guideRNA was obtained after isolation from PAGE. The integrity and full conversion (+568 Da) of the guideRNAs was approved by MALDI-TOF mass analysis. The sequences for the CCX series are 5′-BG-r(UCG GAA CAC CCC XGC ACA GA), with X = U, A, C, G; for the CUX series are 5′-BG-r(UCG GAA CAC CCU XGC ACA GA), with X = U, A, C, G; and for the CXA series varying the counter base of the targeted adenosine are 5′-BG-r(UCG GAA CAC CCX AGC ACA GA), with X = U, A, C, G. The concentrations of all guideRNAs were estimated with an extinction coefficient of 242 mM^−1^cm^−1^ at 260 nm.

### Editing reactions

Editing was performed on 25 μl scale by incubating the respective mRNA (50 nM) with the BG-modified guideRNA (200 nM), and the respective SNAP-ADAR1/2/3 (350 nM) in 75 mM KCl, 25 mM Tris-HCl, 2 mM DTT, 0.75 mM MgCl_2_, pH 8.3 for 3 h, while cycling between 30°C (30 min) and 37°C (30 min). Editing was finished by adding 100 equivalents of a ssDNA oligomer complementary to the respective guideRNA (CCX and CXA series: 5′-d(ATC TGT GCT GGG GTG TTC CGA T); CUX series: 5′-d(ATC TGT GCT AGG GTG TTC CGA T)) and heating to 70°C for 2 min. After reverse transcription with M-MuLV reverse transcriptase (New England Biolabs) and with the reverse primer 5′-d(CAG CGG TGG CAG CAG CCA AC), the cDNAs were purified using spin columns (PCR clean-up kit, Macherey Nagel). Taq polymerase PCR reactions were templated with cDNA, forward primer 5′-d(GCG GAT AAC AAT TCC CCT CTA G) and backward primer 5′-d(CAG CGG TGG CAG CAG CCA AC) to obtain PCR products that were sequenced by Sanger sequencing (MWG Eurofins or LGC Genomics). The yield of edited mRNA was estimated by the relative areas of the guanine trace versus the adenine trace in the abi sequencing trace. Reactions were run both in presence (0.75 mM) or in absence (0 mM) of magnesium as indicated.

## RESULTS AND DISCUSSION

### Controlling editing selectivity with guanosine mismatches

Natural editing substrates present the targeted adenosine either paired with uridine or mismatched with cytosine. In the literature (17) you find hints that RNA substrates that put an adenosine base into mismatch with a purine base are less well edited. With our standard CFP reporter gene (9), carrying a Stop66 (UAG) nonsense mutation centrally in 17 bp guideRNA/mRNA duplex, we tested the effect of the counter base (U, C, A, G) on the editing yield. Whereas U and C gave quantitative yields, editing was less efficient with adenosine and severely hampered with guanosine as the counter base (see Supplementary Figure S1).

**Figure 3.**
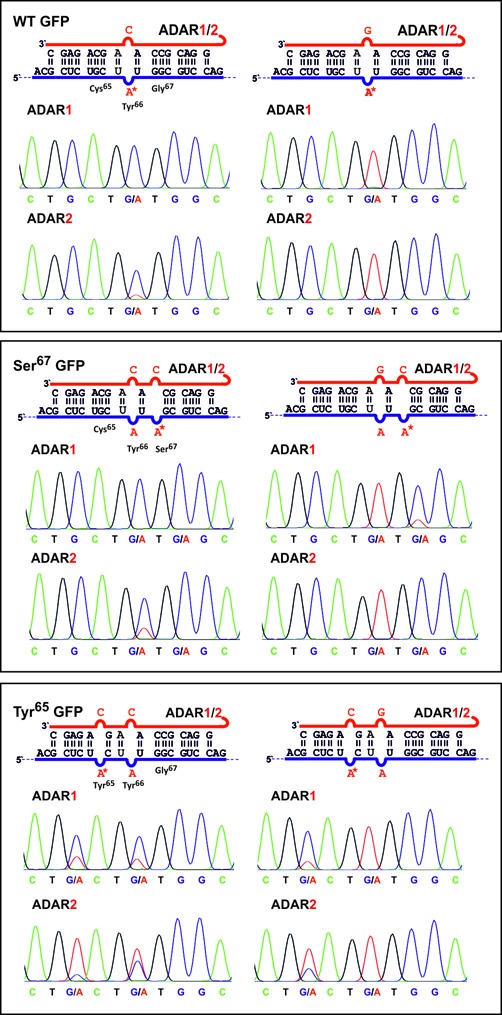
Control of editing selectivity by guanosine mismatching. Mismatching an adenosine base with guanosine completely inhibits editing. This can be exploited to block unwanted off-site editing of the functionally important Tyr66 codon (UAU) in GFP mRNA. G-mismatching allows for the highly selective editing of Ser67 (U AGC) and Tyr65 (UAC) missense mutations in GFP. However, the latter is only efficiently edited with SNAP-ADAR1. The guideRNA is shown in red, the mRNA in blue and the targeted adenosine is marked by an asterisk.

Since directed RNA editing can lead to off-site editing in adenosine-rich sequences (10) we got interested in the possibility to use guanosine mismatching as a simple means to suppress editing at neighboring bases. A suitable test case is the repair of G-to-A missense mutations in GFP in direct vicinity to the functionally essential Tyr66 codon (UAU). As described before (9), two missense mutations, Cys65Tyr (UGC→UAC) and Gly67Ser (U GGC → U AGC), have been generated that both entirely inhibit fluorophore maturation. First, we demonstrated the Tyr66 (UAU) codon to be readily editable when activated by mismatching with cytosine (Figure 3, first panel). Editing at this essential residue completely destroys the fluorescent phenotype, antagonizes the repair of the two other point mutations and thus should be avoided. Indeed, the Tyr66 codon is not edited when the targeted adenosine is mismatched with guanosine (Figure 3, first panel). We then studied the repair of the Ser67 GFP point mutation by editing. We observed nearly quantitative editing at both codons simultaneously when both codons, Ser67 and Tyr66, are put into A/C-mismatches (Figure 3, middle panel). Thus, the gene function is efficiently repaired at the Ser67 codon but immediately destroyed at the Tyr66 codon by over-editing. However, putting the Tyr66 codon in a A/G-mismatch allowed for the nearly quantitative editing of the A/C-mismatched Ser67 codon with both SNAP-ADAR1 and -ADAR2 without any detectable over-editing at Tyr66. Guanosine mismatching was efficient in protecting the Tyr66 codon from over-editing. We expected a similar finding when applying the same strategy to the repair of the Tyr65 missense mutation. Indeed, SNAP-ADAR1 gave access to a highly selective and efficient editing once the Tyr66 codon was protected by an A/G-mismatch (Figure 3, lower panel). In contrast, SNAP-ADAR2 did not accept the doubly mismatched RNA substrate very well. This was true for both double mismatches A/C+A/C and A/C+A/G (Figure 3, lower panel), even though the single A/C-mismatch at Tyr66 was well accepted by SNAP-ADAR2 before (first panel in Figure 3). Thus, editing at the Tyr66 codon can be entirely abolished by mismatching with guanosine; however, the editing yield at the targeted Tyr65 codon remains low.

Taken together, an excellent editing selectivity can be achieved by as simple means as the choice of the counter base. This strategy was even successful when the targeted and the off-site base were separated by only one intervening nucleotide. In contrast to SNAP-ADAR1 that gave reliably high editing yields, this strategy is feasible but less predictable for SNAP-ADAR2. Thus guanosine mismatching well complements other strategies for controlling editing selectivity including positioning of the guideRNA (9) and 2′-O-methylation of the guideRNA (10).

### Editing of difficult codons

**Figure 4.**
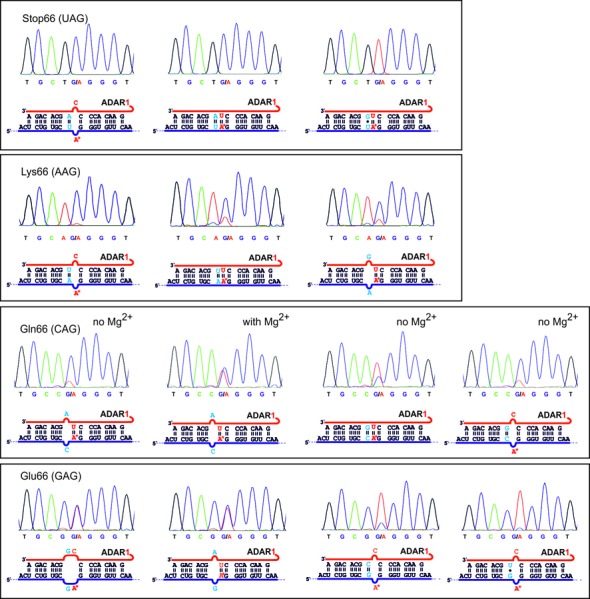
Selection of sequencing results for the codons XA*G, X = U, A, C, G. For every codon, the optimal guideRNA architecture is given first, followed by suboptimal architectures that show the necessity for matching (UAG) or mismatching at the 5′-neighbor X (CAG and GAG) and a notable dependence on magnesium (CAG). Except the three marked examples all editings were performed in presence of 0.75 mM magnesium.

Whereas the sequence context (number of proximate adenosines) may limit editing due to unspecific over-editing, some codons (5′-GAX for instance) are inherently resistant against editing. To activate such codons we systematically tested all conceivable matches and mismatches at the 5′-neighbor for all four codons XAG, X = U, A, C, G. With regard to our findings above, we decided to present the targeted adenosine either in base pair with uridine or in mismatch with cytosine (see Figure 2). Whereas the sequencing traces shown in Figure 4 represent only a selection of particularly interesting guideRNA architectures, the complete set of sequencing traces in presence and absence of magnesium during editing can be found in the Supporting Information (Supplementary Figures S2–S10). This section is organized by discussing the optimal architecture of each of the four codons XAG with X = U, A, C, G.

### Editing of the UAG codon

In accordance to the literature, we found editing of the UAG codon to be particularly efficient; see Table 1 and Figure 4. Both proteins SNAP-ADAR1 and SNAP-ADAR2 achieve quantitative (>80%) conversion. Both domains have similar requirements with respect to the guideRNA/mRNA duplex: quantitative editing requires full complementarity. Only at the targeted adenosine a mismatch with cytosine is accepted (as discussed above). SNAP-ADAR2 does not accept any mismatch at the 5′-neighboring position. In contrast, SNAP-ADR1 is more promiscuous and also accepts pyrimidine/pyrimidine mismatches to some extent, however, with a dramatic loss in editing yield. Also natural editing substrates often present adenosines in mismatch with cytosine, for instance in the R/G-site of the gluR receptor gene (1,2). It was argued in the literature that mismatching the adenosine with cytosine would facilitate the flip-out mechanism to bring the adenosine inside the catalytic pocket (2,18). This brings up the idea of a specific recognition of the orphan counter base similar to that found for the DNA methyltransferase HhaI (19). Unfortunately, until today there is only a crystal structure of the empty deaminase domain of ADAR2 available, thus there is no structural evidence that supports this idea (18).

**Table 1.**
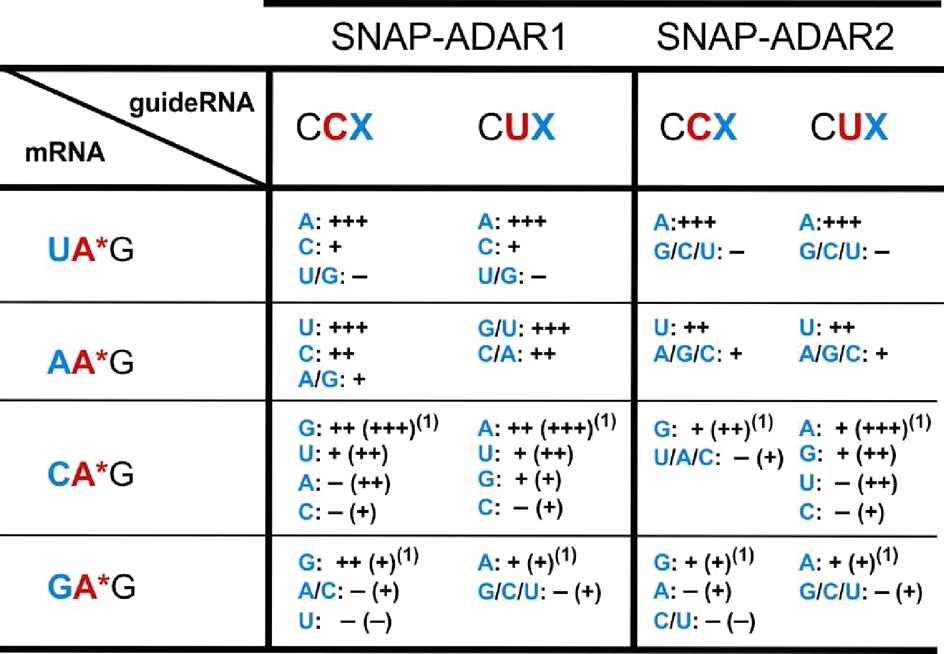
Overview of the results obtained for all 64 possible guideRNA architectures as indicated in Figure 2

The editing yields were estimated from the relative signal of adenosine versus guanosine in the sequencing traces. Since editing yields vary to some extent the yields are reported in four rough categories: − (<15% yield, basically inactive), + (15–50% yield, slight activity), ++ (>50–80% yield, decent activity), +++ (>80% yield, up to quantitative). A selection of sequencing traces is shown in Figure 4. The sequencing traces for all 64 settings are given in duplicate in the Supporting Information (Supplementary Figures S2–S5). The superscript ^**(1)**^ shows that the yields given in brackets were obtained under the same conditions but in absence of magnesium (Supplementary Figures S2–S5).

### Editing of the AAG codon

The global editing analyses reported in the literature (12,20,21) are often restricted to nearly perfectly base-paired transcripts. From their findings and the editing of the UAG codon above, one could expect that the AAG codon has similar requirements but may give a reduced editing yield or selectivity. Indeed, SNAP-ADAR2 prefers the A/C mismatch at the targeted site and full complementarity around. Compared to the UAG codon, however, the yield reaches only half conversion and low-level editing is observed for various 5′-mismatches. In contrast to SNAP-ADAR2, -ADAR1 edits the AAG codon in many sequence contexts decently (>50%) and reaches up to quantitative yield (>80%) if the targeted adenosine is mismatched with cytosine and if full complementarity is retained around. If the targeted adenosine is matched with uridine, every possible mismatch gives yields of >50%. This promiscuity is clearly distinct from the editing of the UAG codon. Over-editing occurs at the 5′-neighboring adenosine base to a low extent for both SNAP-ADAR1 and -ADAR2. The low amount of over-editing may be due to the deactivating effect of the 5′-neighboring cytosine base (5′-CAAG). However, in accordance to our experiments above, the level of over-editing at this site is stronger when that base is mismatched with a pyrimidine rather than with guanosine. Thus the acceptance for an A/G mismatch at this position could potentially be used to steer selectivity in a sequence context providing a more reactive codon at the site of over-reaction as 5′-UAAG, for instance. The results show that optimizing the guideRNA for a specific sequence and ADAR domain requires very specific knowledge that cannot be derived from global analyses (12). Obviously, not only 5′-neighboring base pairs but also 5′-neighboring mismatches can positively influence the outcome of an editing reaction. We also tested the influence of physiological (0.75 mM) versus no magnesium on editing (compare Supplementary Figures S3 and S8). However, the effect was very little and occurred in both directions (activation and deactivation). The biggest effect was found for the optimal guideRNA (CCU, ADAR1) which worked better with magnesium (nearly quantitative, >80%) than without (≈50% yield).

### Editing of the CAG codon

The situation for the editing of the CAG codon is the most complex of all. First, the editing yields strongly and systematically depend on the magnesium concentration (compare Supplementary Figures S4 and S9). In presence of magnesium cations, only SNAP-ADAR1 but not SNAP-ADAR2 gives yields of >50%. However, in absence of magnesium, both SNAP-ADAR1 and -ADAR2 can obtain up to quantitative yields (>80%) with the same preferred guideRNAs: if the targeted adenosine is mismatched with cytosine then a C/G match is preferred as the 5′-neighbor. In contrast, when the targeted adenosine is matched with uridine then a C/A mismatch is preferred. One could speculate if the editing of the targeted adenosine is influenced by the editing of the neighboring adenosine in the guideRNA. However, also a C/G base pair 5′ to the targeted adenosine is accepted to some extent. Both enzymes prefer the targeted adenosine to be base-paired with uridine. Full complementarity is the situation at the Q/R-site of the gluR gene (1). We can reproduce here that SNAP-ADAR2 edits this codon better than SNAP-ADAR1 (12) if a fully complementary guideRNA (CUG) is applied. Taken together, a huge variety of matches and mismatches is accepted at the 5′-neighboring position, but one 5′-mismatch reliably gave the worst editing yields in all CAG settings. That was the C/C mismatch. With respect to the promiscuity the situation is similar as seen for the AAG codon and different from the UAG codon.

### Editing of the GAG codon

To our experience, adenosines that lie downstream of a guanosine residue are protected from editing. In many sequences, we benefit from this because we do not need to protect such adenosine residues from unwanted over-editing. However, to reprogram genetic information, it would be desirable to overcome this limitation that affects one-fourth of the potentially editable codons. In particular, premature stop codons are closely linked to many diseases due to the complete removal of the transcript caused by nonsense-mediated decay (22,23). However, repairing the abundant opal STOP codon (UGA) that is obtained by a single point mutation from Trp (UGG), Arg (CGA, AGA), or Gly (GGA) is currently inaccessible by RNA editing. One could refine the deaminase domain by means of laboratory evolution to better accept the 5′-GA codon (24,25). Due to the limited structural knowledge about the binding of the substrate duplex, this appears difficult but feasible to some extent (24). Another strategy is to activate the substrate by optimizing the secondary structure of the guideRNA/mRNA duplex.

Applying the typical substrate architecture with a fully complementary RNA duplex and the targeted adenosine either in a base pair with uridine or in mismatch with cytosine gave no editing yield above 15%. However, editing of the GAG codon is substantially activated up to ≈50% yield by putting a G/G or G/A mismatch 5′ to the targeted adenosine. Both counter bases C and U are accepted. However, the 5′ mismatch is mandatory. Already a G/U wobble base pair is completely inactive. In this case, the findings are similar for SNAP-ADAR1 and -ADAR2, but with -ADAR2 giving lower editing yields. Again, one could speculate if the editing of the guideRNA in neighborhood to the targeted adenosine interferes with the editing at the target site. That both codons, GAG and CAG, are most efficiently edited with the same guideRNA (CUA) could potentially become limiting in later *in-vivo* applications when those codons need to be discriminated in very similar sequence contexts. However, taken together we have demonstrated that particular secondary structures can even activate the most difficult codons. In a global analysis of A-to-I editing of human Alu repeats, it was shown before that positioning of adenosine bases inside interior loops with helical structures at both ends can activate editing (26). However, it is impossible to explain or deduce the optimal guideRNA architecture for a given target sequence based on such global analyses. Different from the CAG codon, we find a minor dependency on the magnesium concentration. Reactions with optimal guideRNAs are slightly impaired, whereas formerly unproductive guideRNA architectures gave little editing (≈15% yield) in absence of magnesium (compare Supplementary Figure S5 and S10).

### Editing with ADAR3

Since no ADAR3 substrate is known, we tested all four mRNAs containing the 5′-XAG codon (X = U, A, C, G) with the four guideRNAs (5′-BG-r(UCG GAA CAC CCC **X**GC ACA GA), **X** = U, A, G, C) that put the targeted base in mismatch with cytosine in absence of magnesium. For all 16 settings no detectable conversion was found, demonstrating the lack deamination activity of SNAP-ADAR3 (Supplementary Figure S6). To rule out that SNAP-ADAR3 requires magnesium or uridine-paired adenosine as a substrate, we additionally repeated the six editing settings that gave optimal results with SNAP-ADAR1 and -ADAR2 in presence of 0.75 mM magnesium. Again, no conversion over background was observed (Supplementary Figure S7). Even though we can only confirm the described catalytical incompetence of ADAR3 (13), the findings show that the editing results obtained with SNAP-ADAR1 and -ADAR2 are not corrupted by endogenous editing activity from the yeast extract.

## CONCLUSION

Redirecting RNA editing activity is a promising tool to alter gene activity in various ways. On the route toward application one has to learn how to activate inherently inactive codons and how to control unwanted off-site editing in adenosine-rich sequence contexts. We demonstrate here that optimizing the guideRNA allows to improve both unwanted off-site editing and activation of difficult codons.

Editing at off-site adenosine residues is efficiently controlled by mismatching with guanosine. This strategy complements our earlier attempts to control over-editing by guideRNA positioning (9) and 2′-O-methylation (10). Thus, the role of the guideRNA is more than simple addressing. The secondary structure of the guideRNA/mRNA duplex controls the outcome of an editing reaction with respect to editing selectivity and efficiency. Referring to the latter, we could now demonstrate that difficult codons can be activated by unprecedented secondary structures. However, optimal guideRNA architectures have to be explored for any given codon/deaminase pair and cannot be predicted from the rules that were obtained from analysis of global editing site selection (12,25). Important parameters are the counter base and the 5′-neighbor. Whereas some codons, as UAG, strictly require a matching base pair at the 5′-site, other codons strictly require a mismatch (GAG), and in some situations both are accepted. For some codons we also find clear differences for the deaminase domain. Whereas the UAG and CAG codons were activated by both SNAP-ADAR1 and -ADAR2, the AAG and GAG codons were much better edited by SNAP-ADAR1. Similarly, we could recently repair the F5 Leiden polymorphism only with SNAP-ADAR2, not with -ADAR1 (10). Due to a lack of structural information, one can only speculate about the molecular basis for this specific behavior. Obviously, the stability of the 5′-neighboring base pair is important for efficient editing. Maybe flipping out the adenosine requires the weakening or unwinding of this base pair and thus suffers from the presence of the strong G/C base pairs at this site (24). However, it remains difficult to explain why the weaker G/U wobble base pair or a mismatch is accepted in some cases but not in others. In particular for the CAG codon and to some lesser extent also for GAG, we find a dependency on magnesium concentration. The absence of magnesium slightly activated many unreactive codons and the promiscuity of both SNAP-ADAR1 and -ADAR2 seems to be increased. Maybe magnesium stabilizes the G/C base pair at the 5′-position too strongly to allow for efficient base flipping. Anyway, *in vitro*, the magnesium concentration can be optimized in order to activate target codons or deactivate non-targeted ones. Since magnesium is present inside the cell in concentrations similar to what we have applied (0.75 mM), we consider these experiments to be more relevant for the cellular application (11).

Our results demonstrate that redirecting RNA editing is not limited to the subset of codons we had surveyed in our initial study (9). Three of four codons investigated in this study are editable to a high degree (≥80%), and even the difficult GAG codon was editable to a substantial amount. This makes us confident that an artificial editing machinery could once be used to manipulate protein and RNA function without too many sequence restrictions.

## SUPPLEMENTARY DATA

Supplementary Data is available at NAR Online.

## FUNDING

Deutsche Forschungsgemeinschaft [STA 1053/3-1]; Fonds der Chemischen Industrie (Sachkostenzuschuss); University of Tübingen. Funding for open access charge: Deutsche Forschungsgemeinschaft [STA 1053/3-1]; University of Tübingen.

*Conflict of interest statement*. None declared.

## Supplementary Material

SUPPLEMENTARY DATA
