# Supplementary material for: Optimal guideRNAs for re-directing deaminase activity of hADAR1 and hADAR2 in trans
Source: Nucleic Acids Res. 2014 Apr 15;42(10):e87. doi: 10.1093/nar/gku272 (PMC4041445; doi:10.1093/nar/gku272)
Supplement: SUPPLEMENTARY DATA [file supp_42_10_e87__index.html]

Optimal guideRNAs for re-directing deaminase activity of hADAR1 and hADAR2 in trans — Optimal guideRNAs for re-directing deaminase activity of hADAR1 and hADAR2 in trans — SUPPLEMENTARY DATA 

# Optimal guideRNAs for re-directing deaminase activity of hADAR1 and hADAR2 in *trans*

## SUPPLEMENTARY DATA

**Files in this Data Supplement:**

- SUPPLEMENTARY DATA
