# Supplementary material for: Optimal guideRNAs for re-directing deaminase activity of hADAR1 and hADAR2 in trans
Source: Nucleic Acids Res. 2014 Apr 15;42(10):e87. doi: 10.1093/nar/gku272 (PMC4041445; doi:10.1093/nar/gku272)

Supporting Information

Optimal guideRNAs for Re-directing Deaminase Activity of hADAR1 and hADAR2 in trans

Marius F. Schneider, Jacqueline Wettengel, Patrick C. Hoffmann and Thorsten Stafforst

Sequencing results

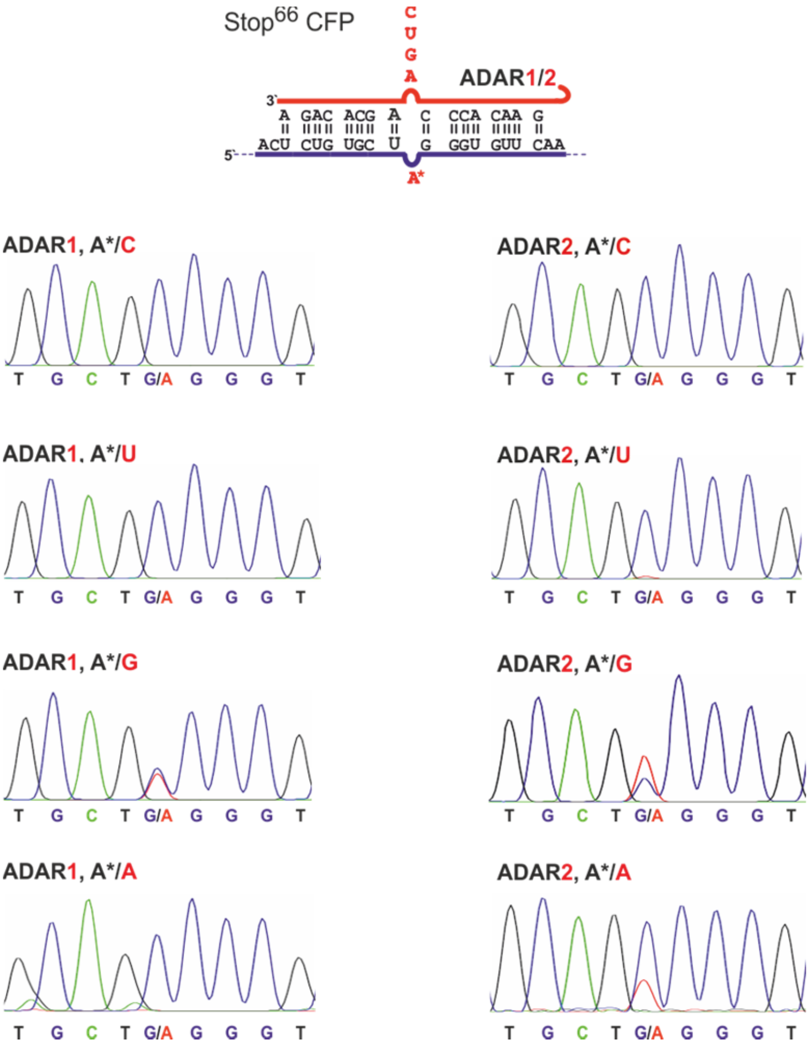

Figure S1. *Effect of the counter base in our standard Stop66 CFP transcript. The adenosine base (A*) of the Stop66 codon (UA*G) was either paired with uridine or mismatched with cytosine, guanosine or adenosine by incubating the mRNA with one of the four BG-modified guideRNAs 5´-BG-r(UCG GAA CAC CC**X** AGC ACA GA), with **X** = C, U, G, or A. The standard editing conditions (concentrations, editing times, etc.) including 0.75 mM magnesium have applied as described in the method part of the manuscript.*

Figure S2.1 *Sequencing traces for the editing of the* ***UAG codon*** *under standard conditions including 0.75 mM magnesium as described in the method part of the manuscript.*

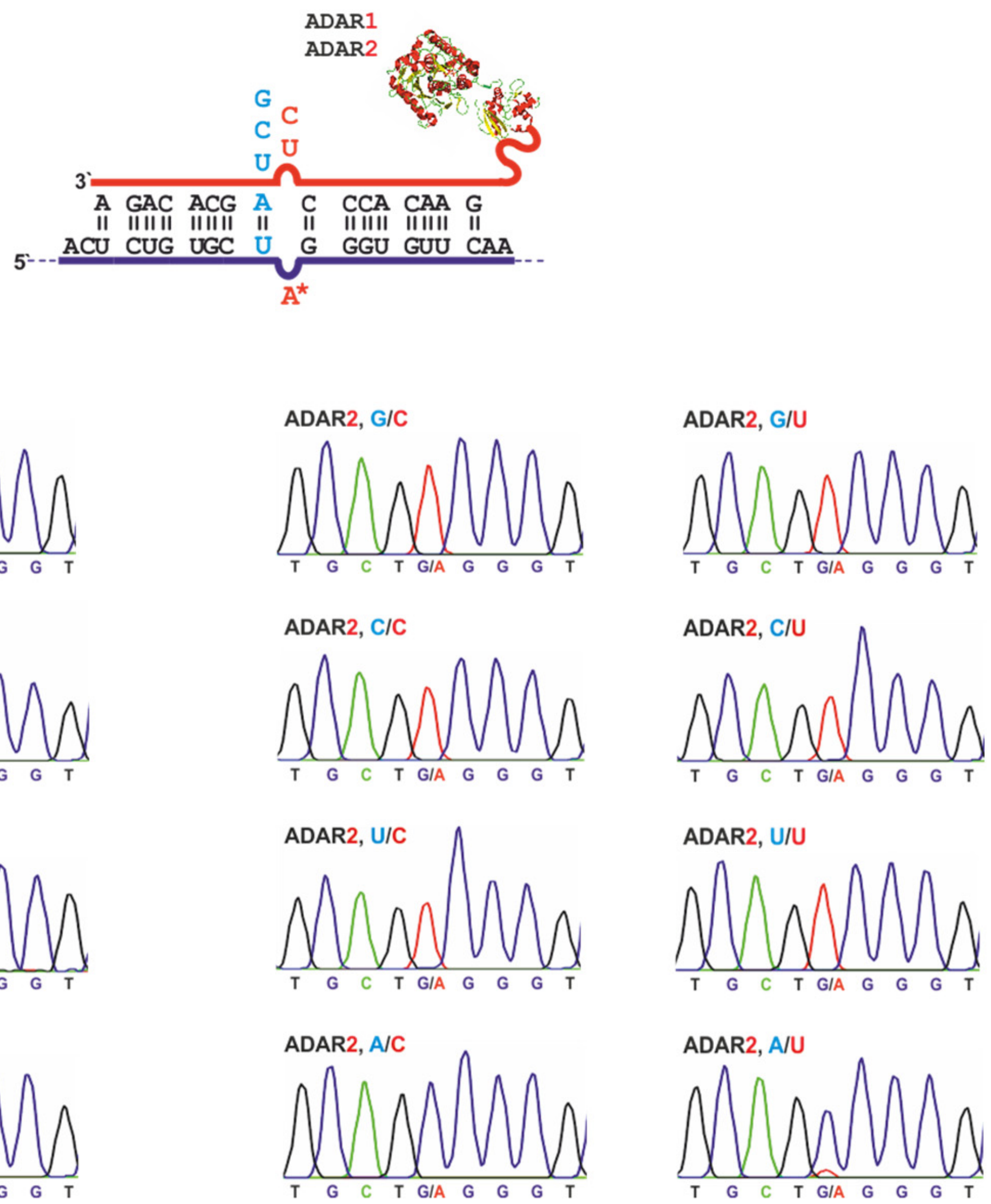

Figure S2.2 *Replicate of experiments shown in Figure S2.1, editing of the* ***UAG codon*** *under standard conditions including 0.75 mM magnesium.*

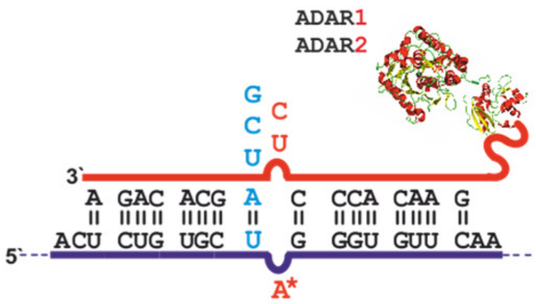

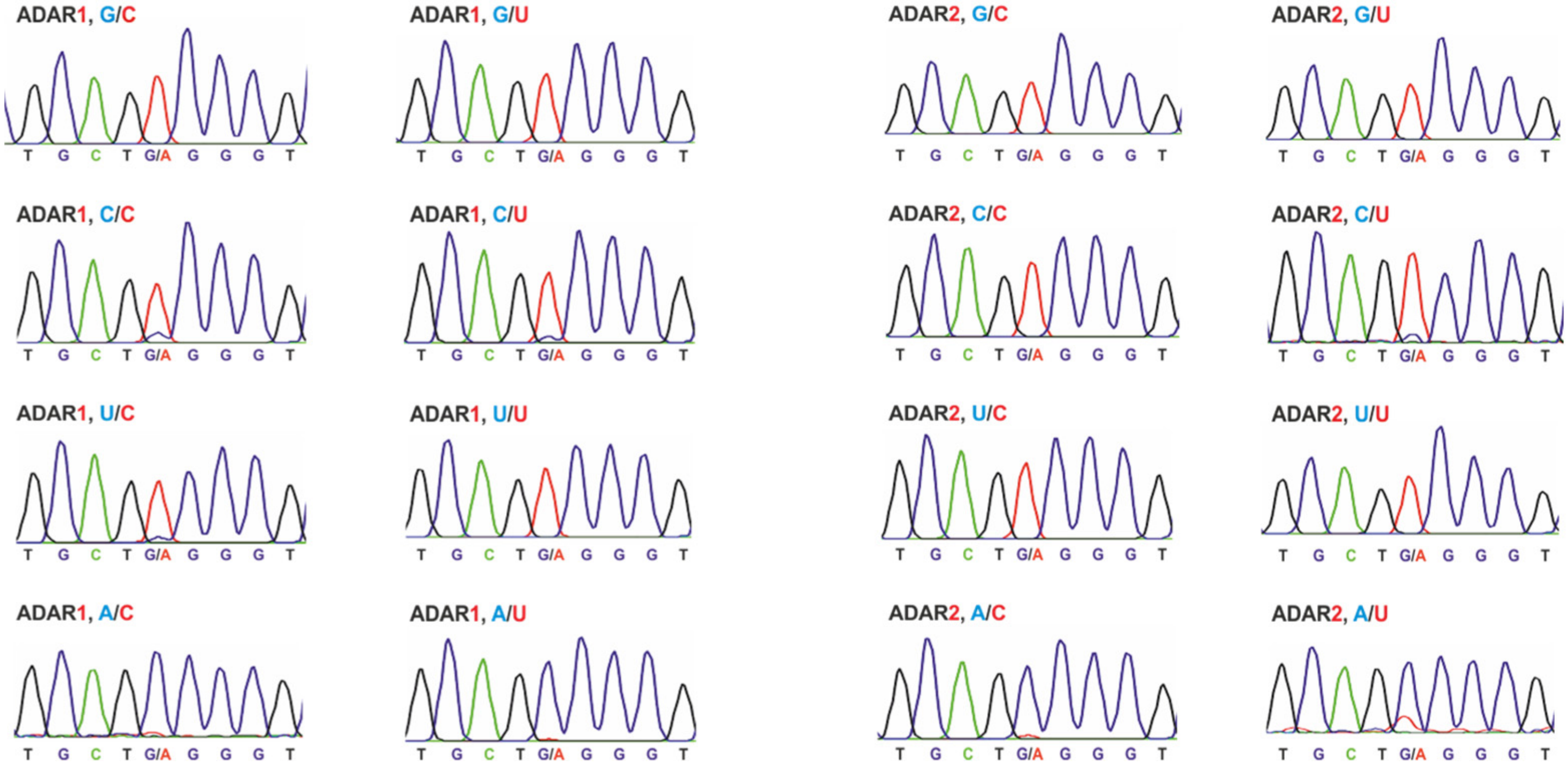

Figure S3.1. *Sequencing traces for the editing of the* ***AAG codon*** *under standard conditions including 0.75 mM magnesium as described in the method part of the manuscript.*

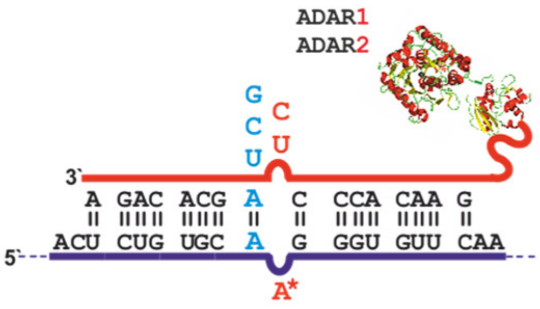

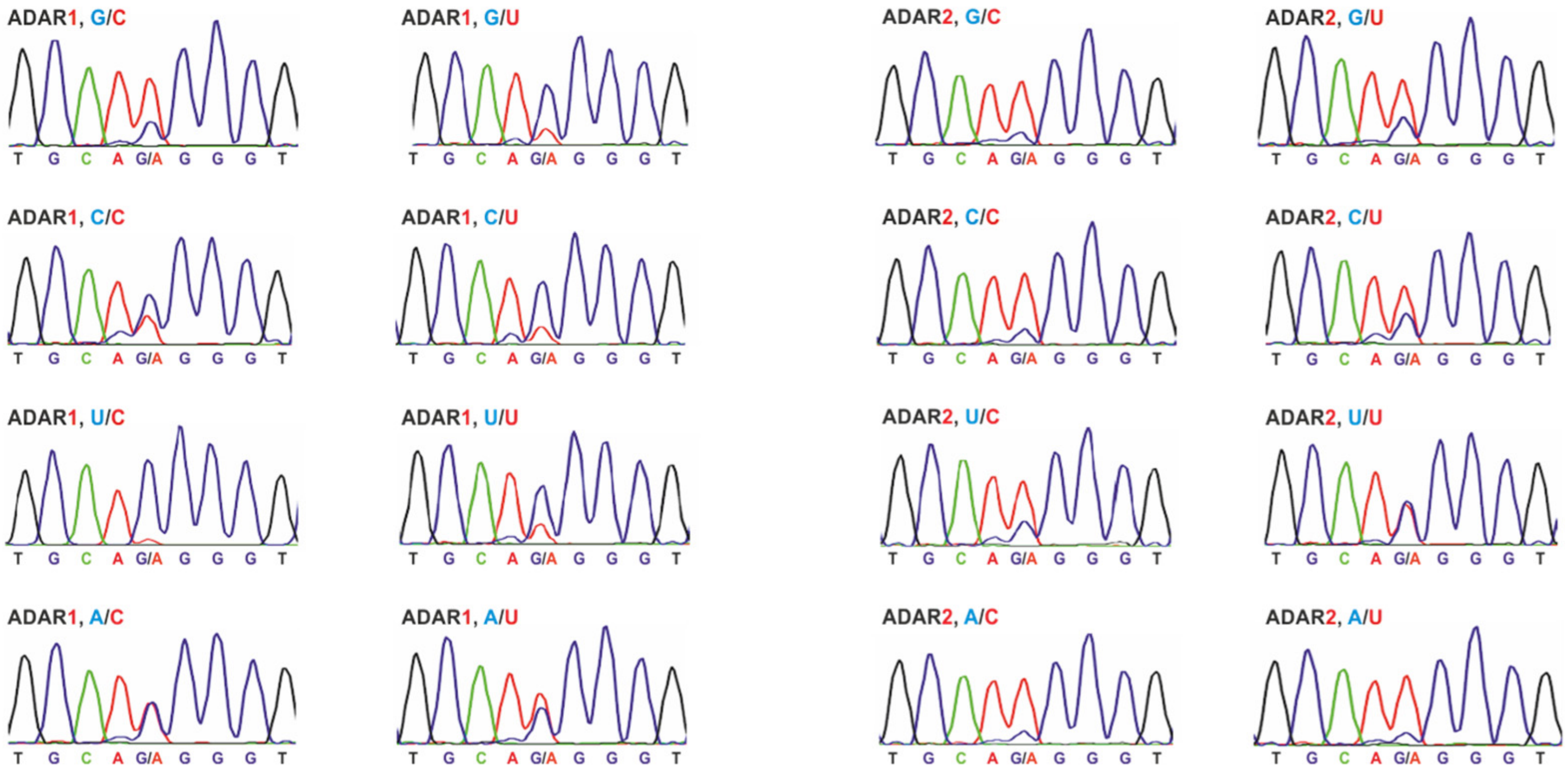

Figure S3.2. *Replicate of the editing of the* ***AAG codon*** *with 0.75 mM magnesium as in Figure S3.*

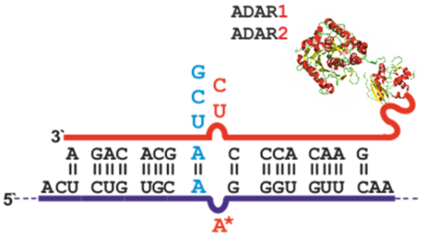

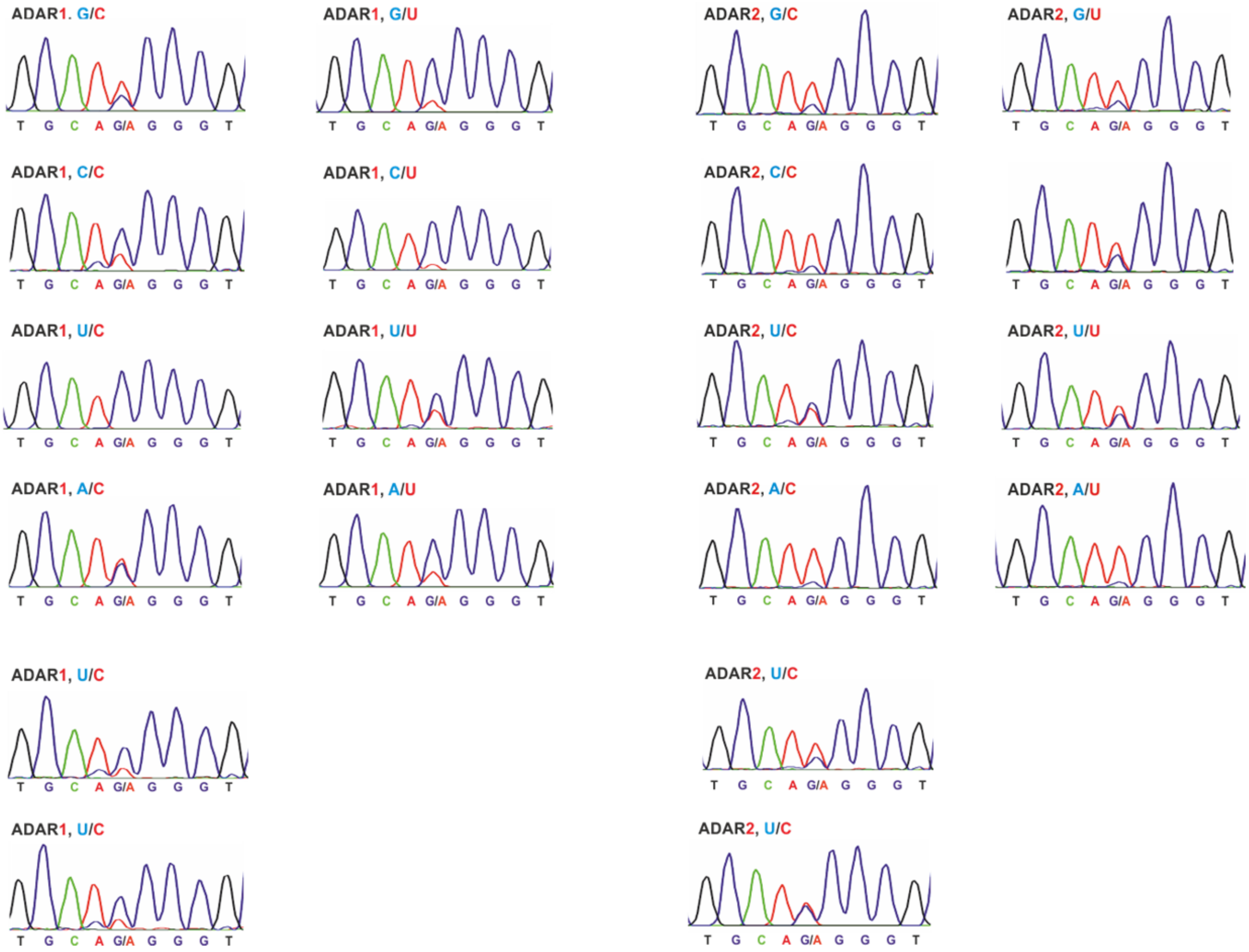

Figure S4.1. *Sequencing traces for the editing of the* ***CAG codon*** *under standard conditions including 0.75 mM magnesium as described in the method part of the manuscript.*

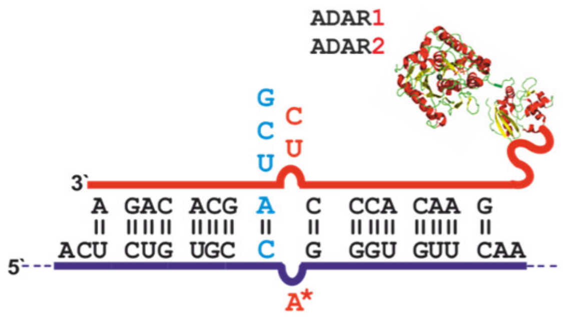

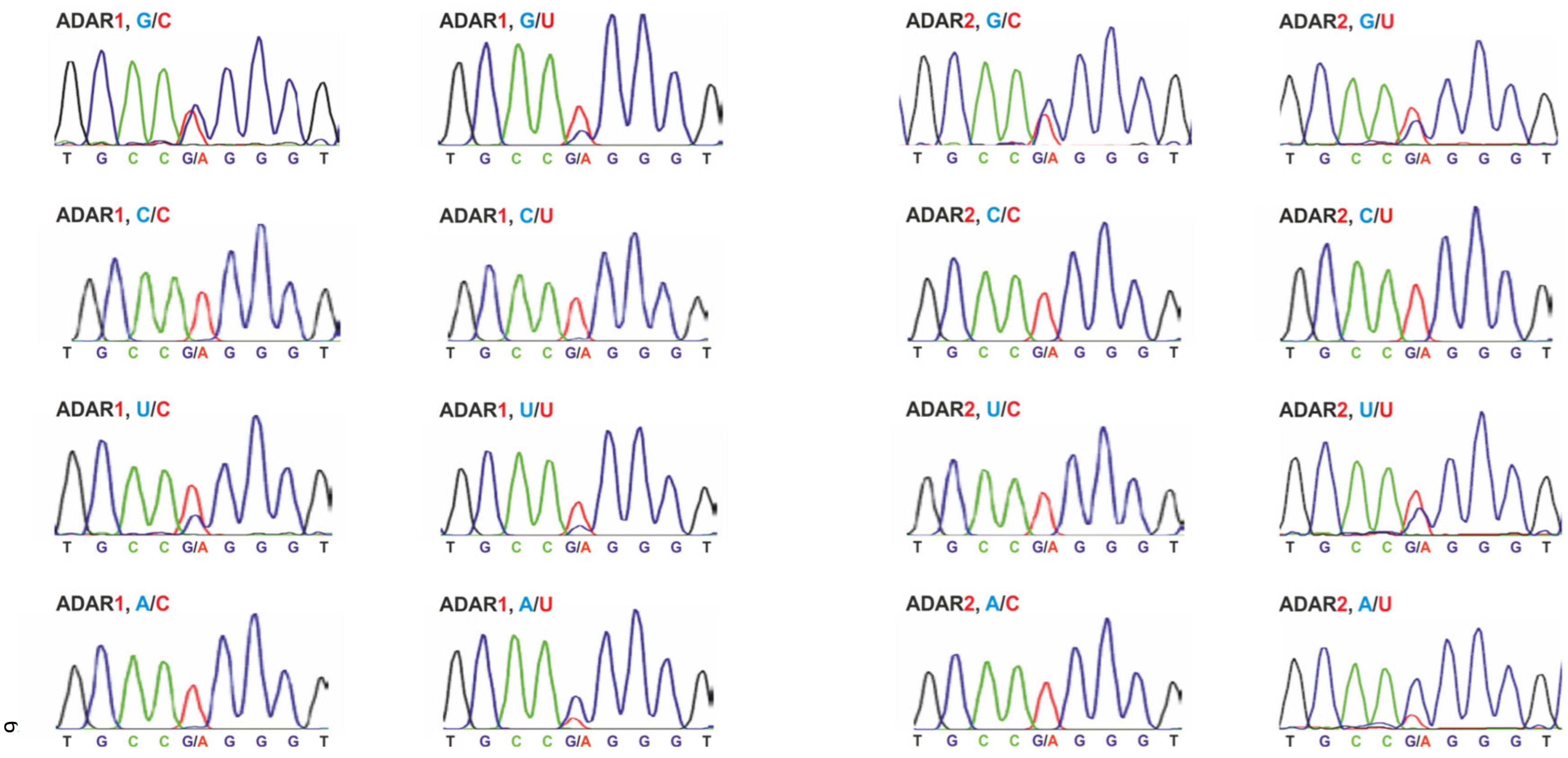

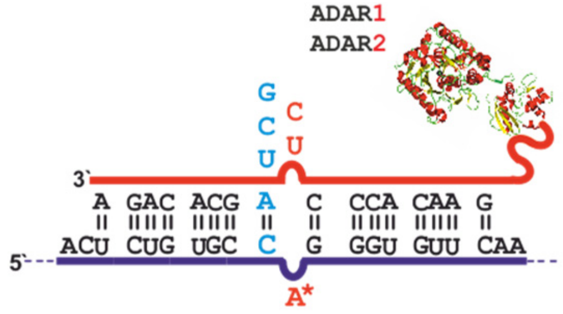

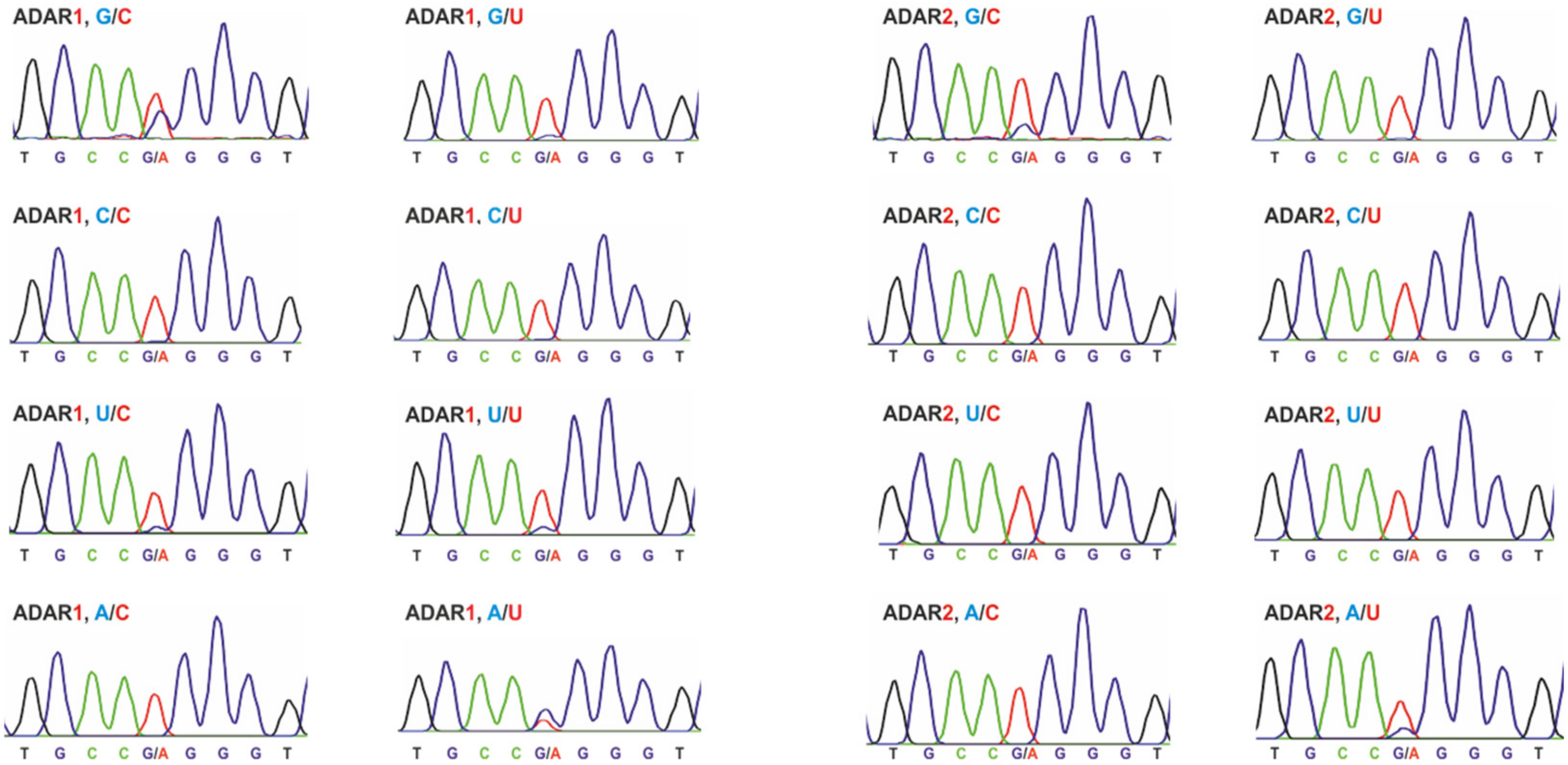

Figure S4.2. *Replicate for the editing of the* ***CAG codon*** *with Mg as described in Figure S4.1.*

Figure S5.1. *Sequencing traces for the editing of the* ***GAG codon*** *under standard conditions including 0.75 mM magnesium as described in the method part of the manuscript.*

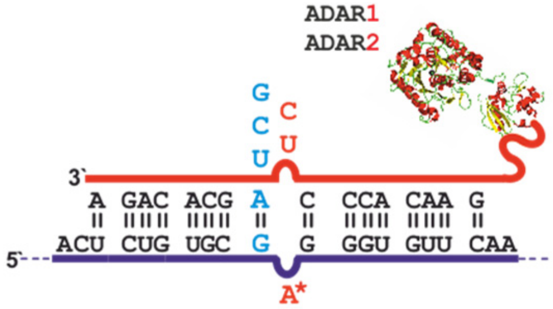

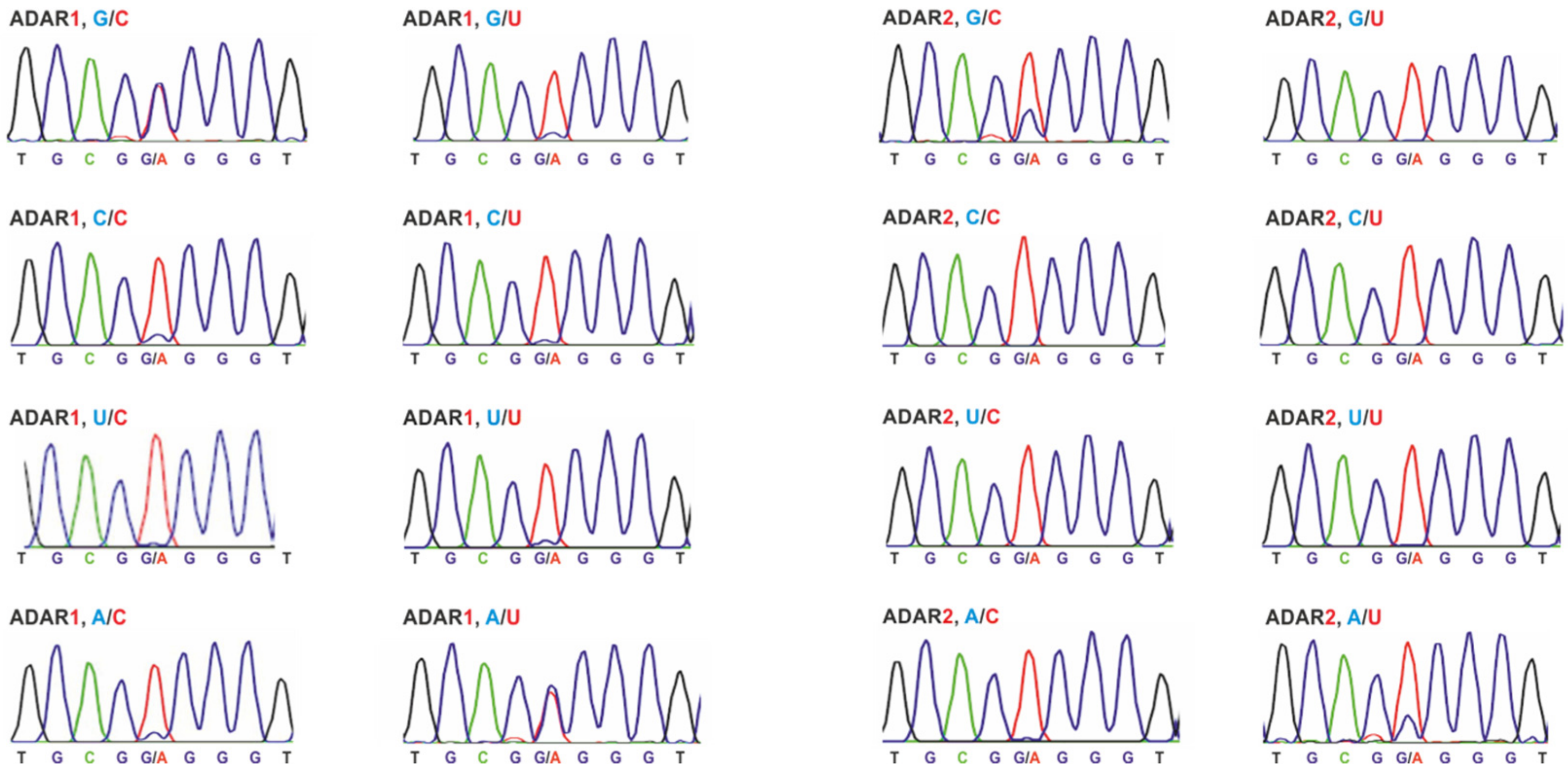

Figure S5.2. *Replication of the editing of the **GAG codon** shown in Figure S5 under standard conditions including 0.75 mM magnesium.*

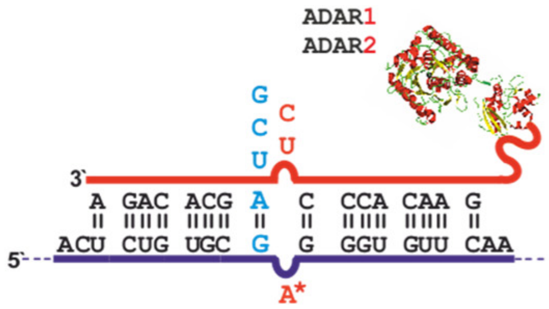

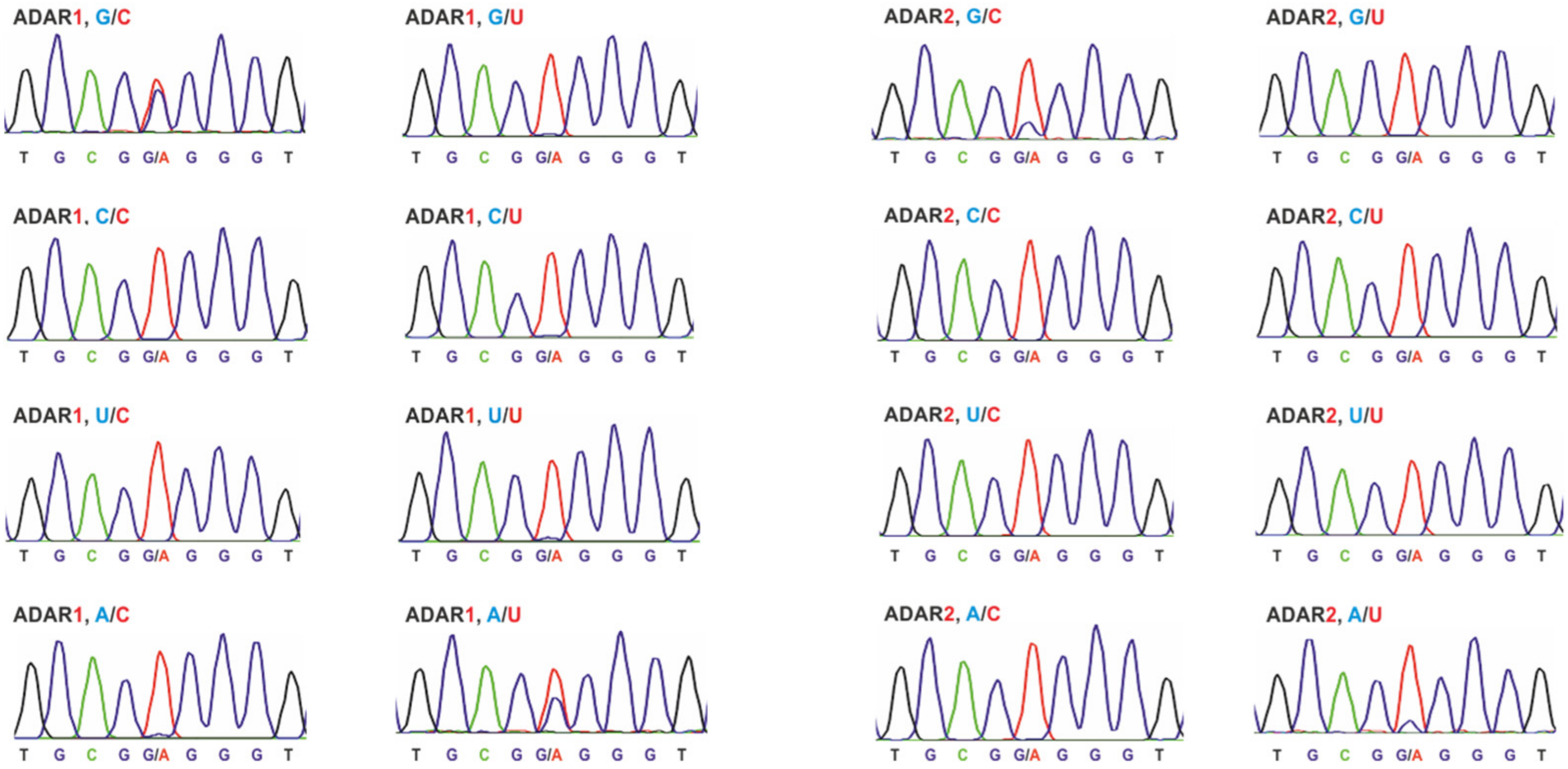

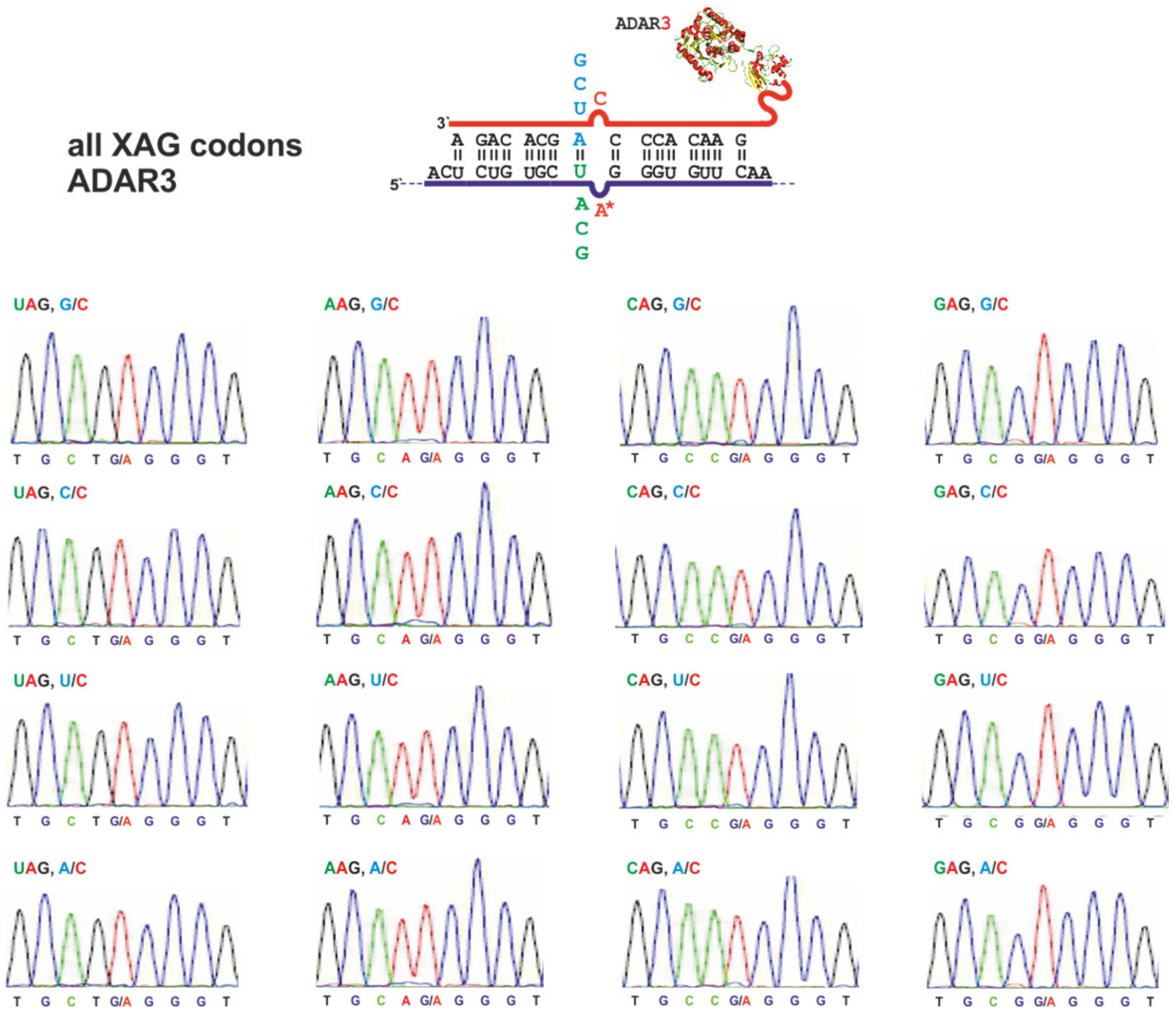

Figure S6. *Sequencing traces for the editing of all four* ***XAG codons*** *(X = U, A, C, G) with SNAP-****ADAR3*** *in presence of one of the four guideRNAs putting the targeted adenosine into A/C mismatch. Editing was done under standard conditions but in the absence of magnesium as described in the method part of the manuscript.*

Figure S7. *Sequencing traces for the editing of all four* ***XAG codons*** *(X = U, A, G, C) with SNAP-****ADAR3*** *and a few selected guideRNAs under standard conditions including 0.75 mM magnesium as described in the method part of the manuscript.*

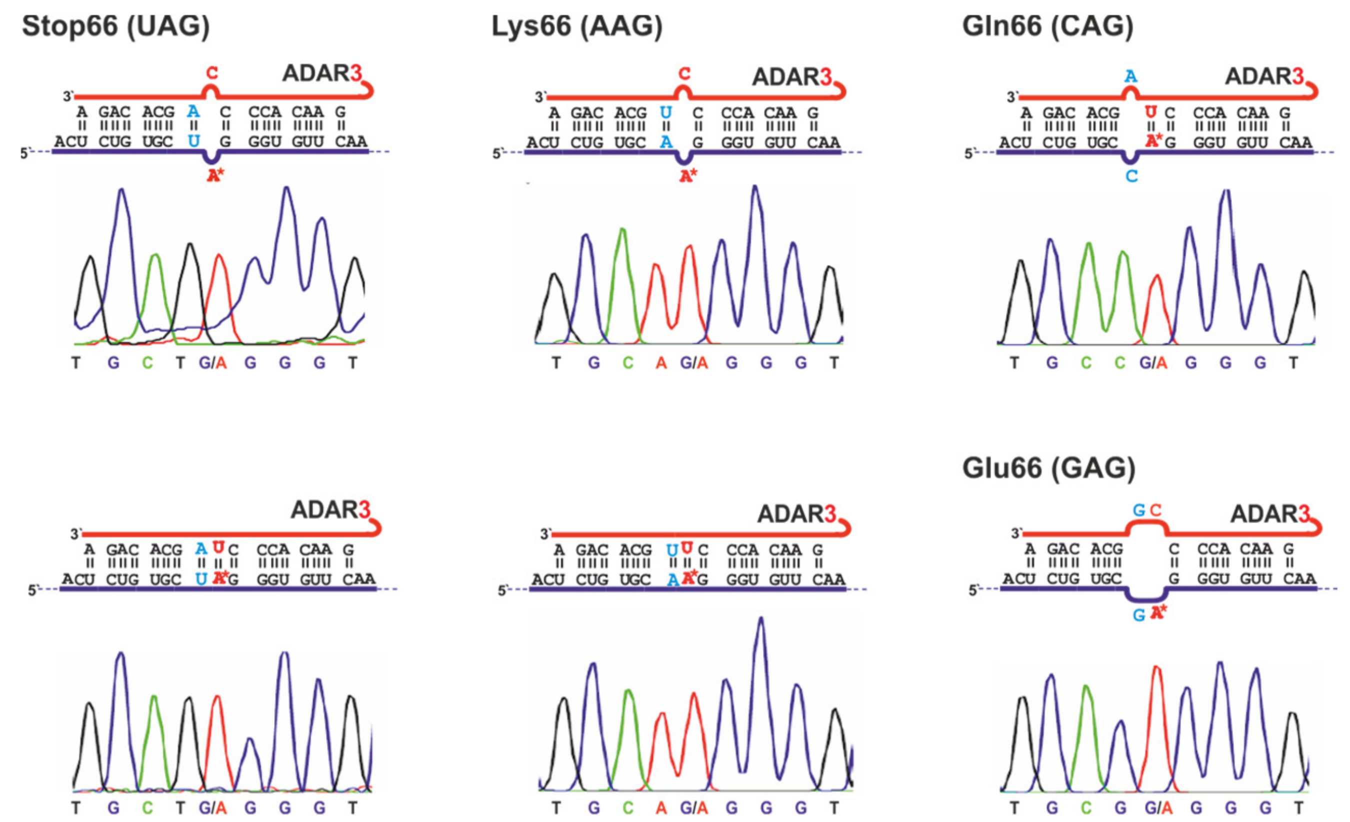

Figure S8. *Sequencing traces for the editing of the **AAG codon** under standard conditions in **absence** of magnesium as described in the method part of the manuscript. All other conditions were kept constant compared to the experiments shown in Figure S3.*

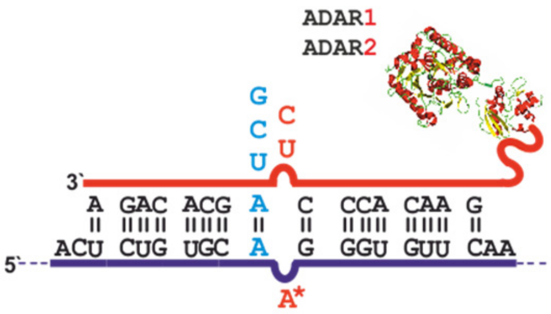

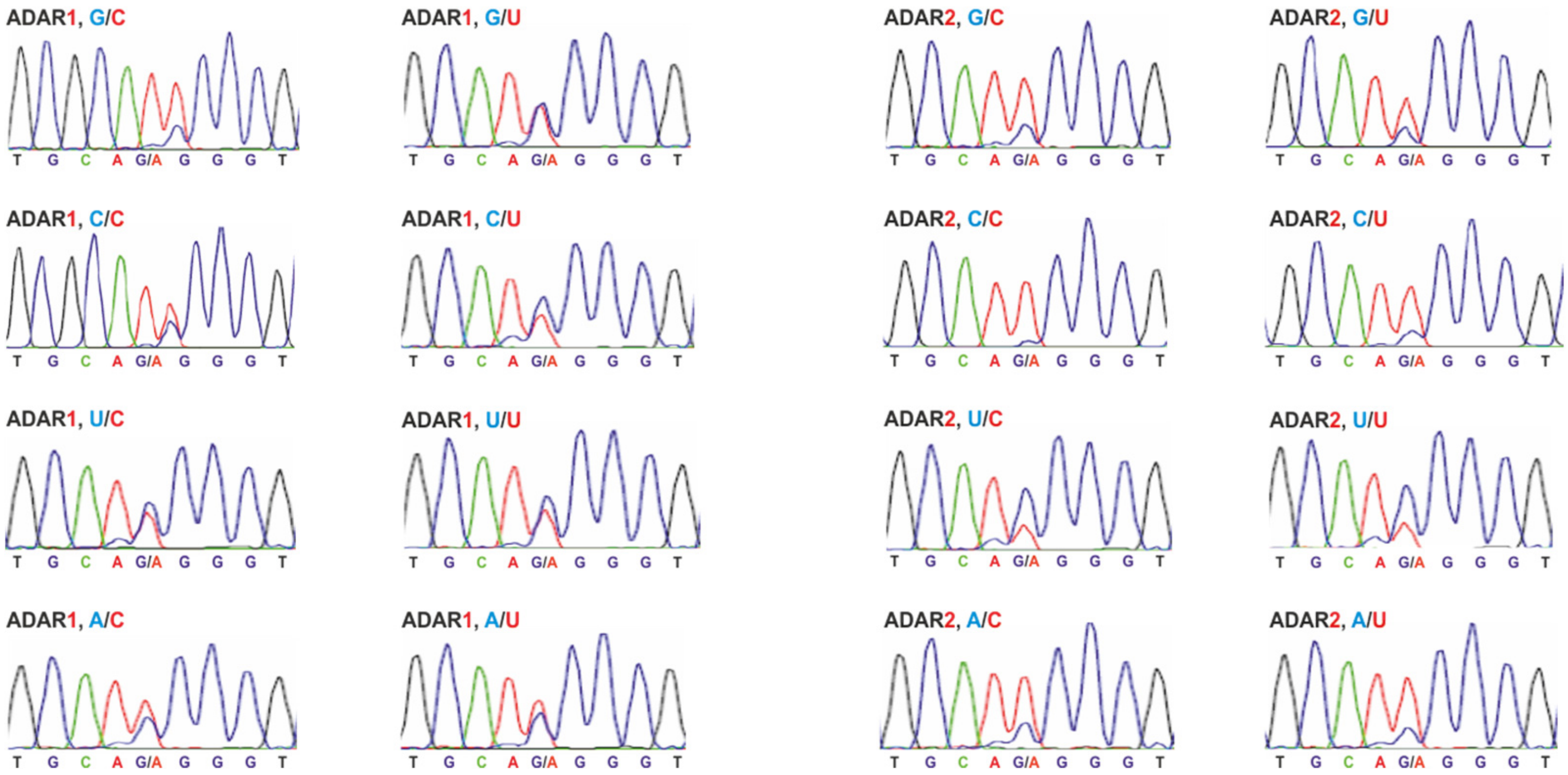

Figure S9.1. *Sequencing traces for the editing of the* ***CAG codon*** *under standard conditions in* ***absence*** *of magnesium as described in the method part of the manuscript. All other conditions were kept constant compared to the experiments shown in Figure S4.*

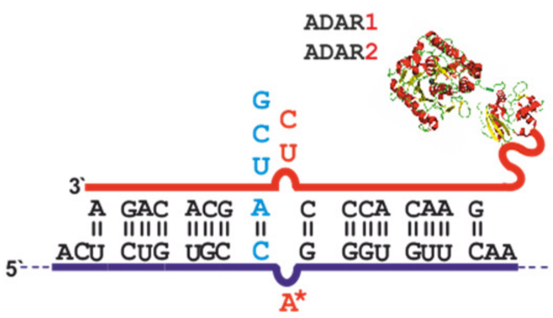

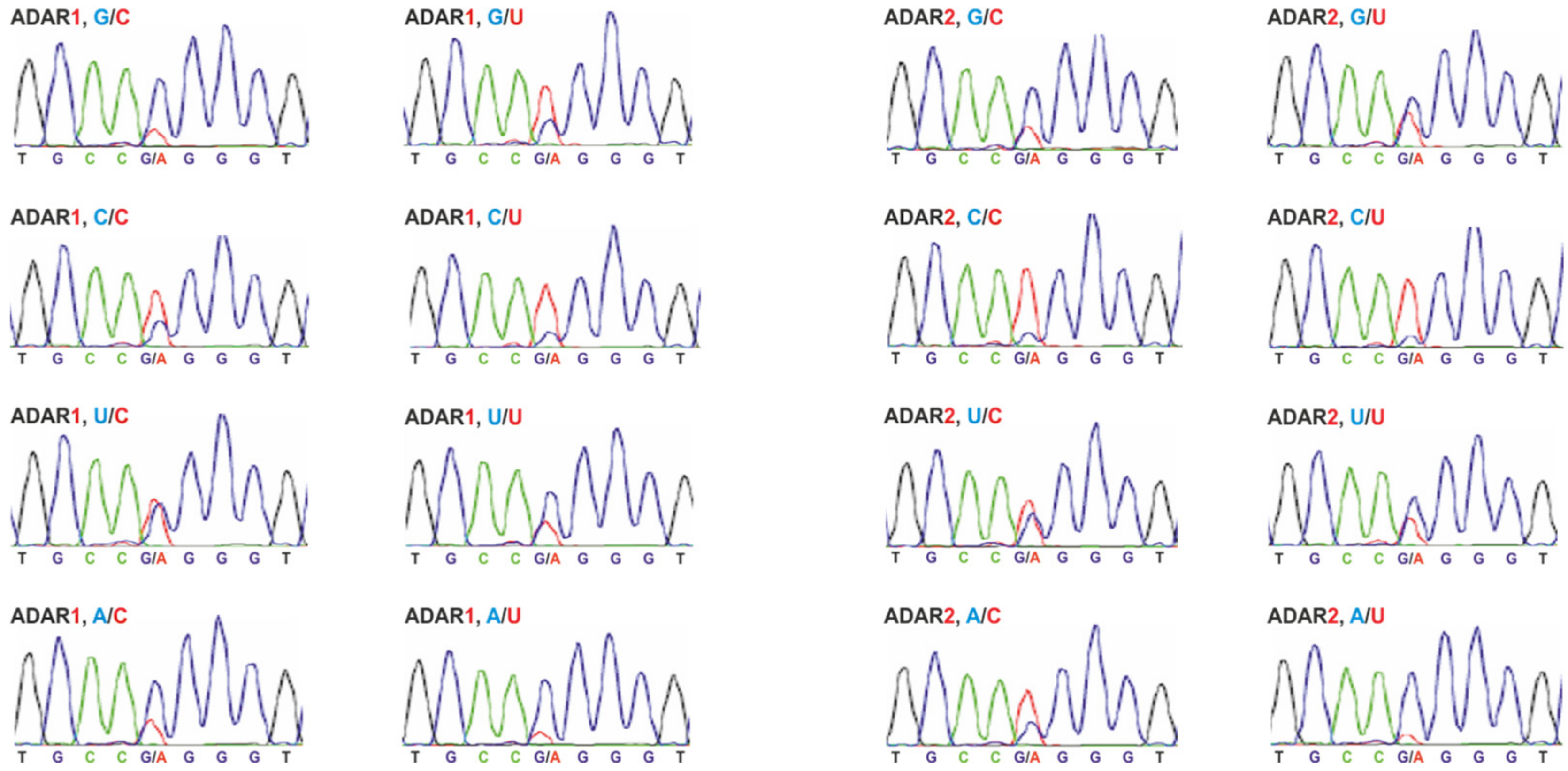

Figure S9.2. *Replicate of editing of the* ***CAG codon*** *under standard conditions in* ***absence*** *of magnesium as described Figure S9.1*

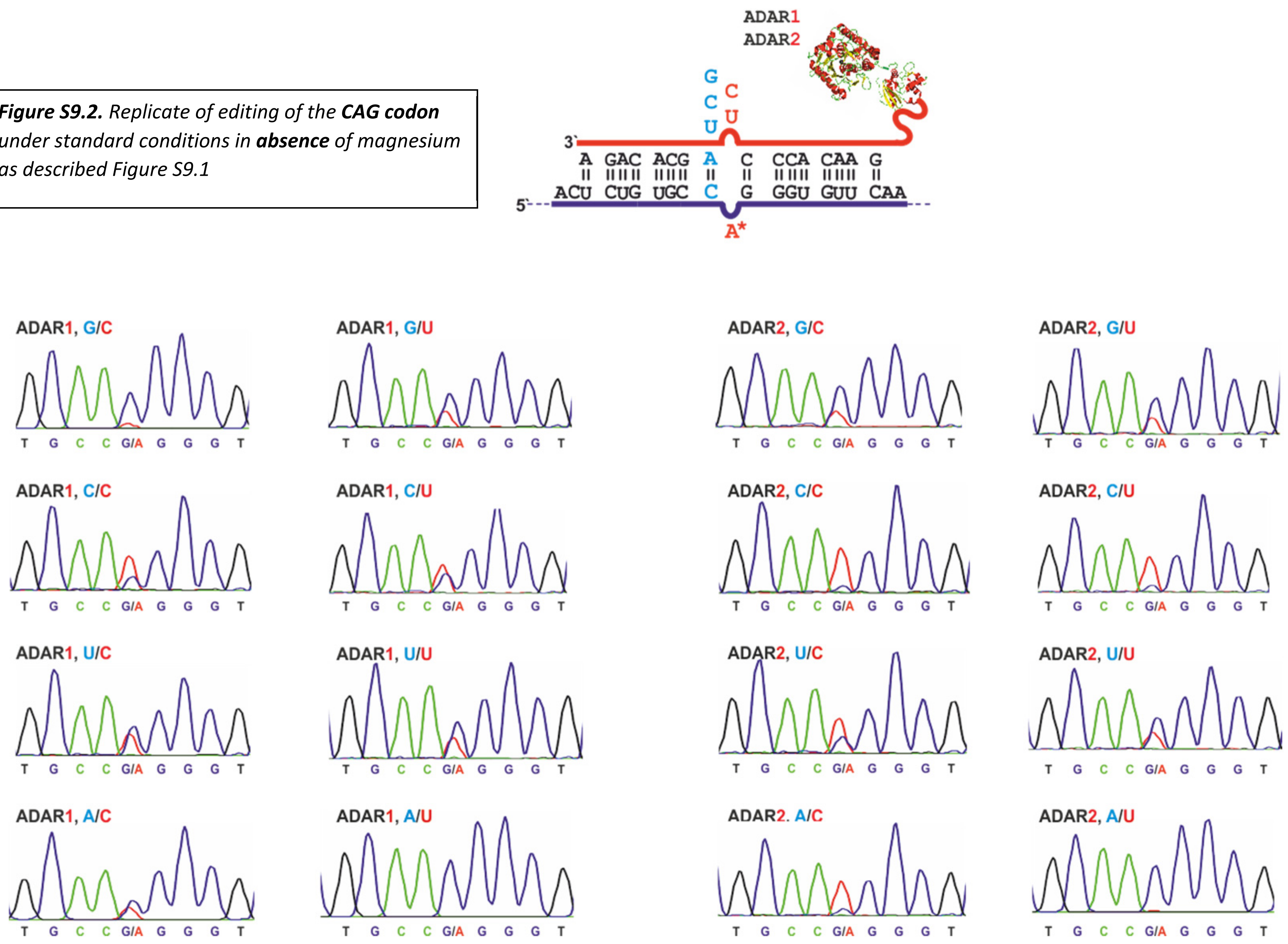

Figure S10. *Sequencing traces for the editing of the* ***GAG codon*** *under standard conditions in* ***absence*** *of magnesium as described in the method part of the manuscript. All other conditions were kept constant compared to the experiments shown in Figure S5.*

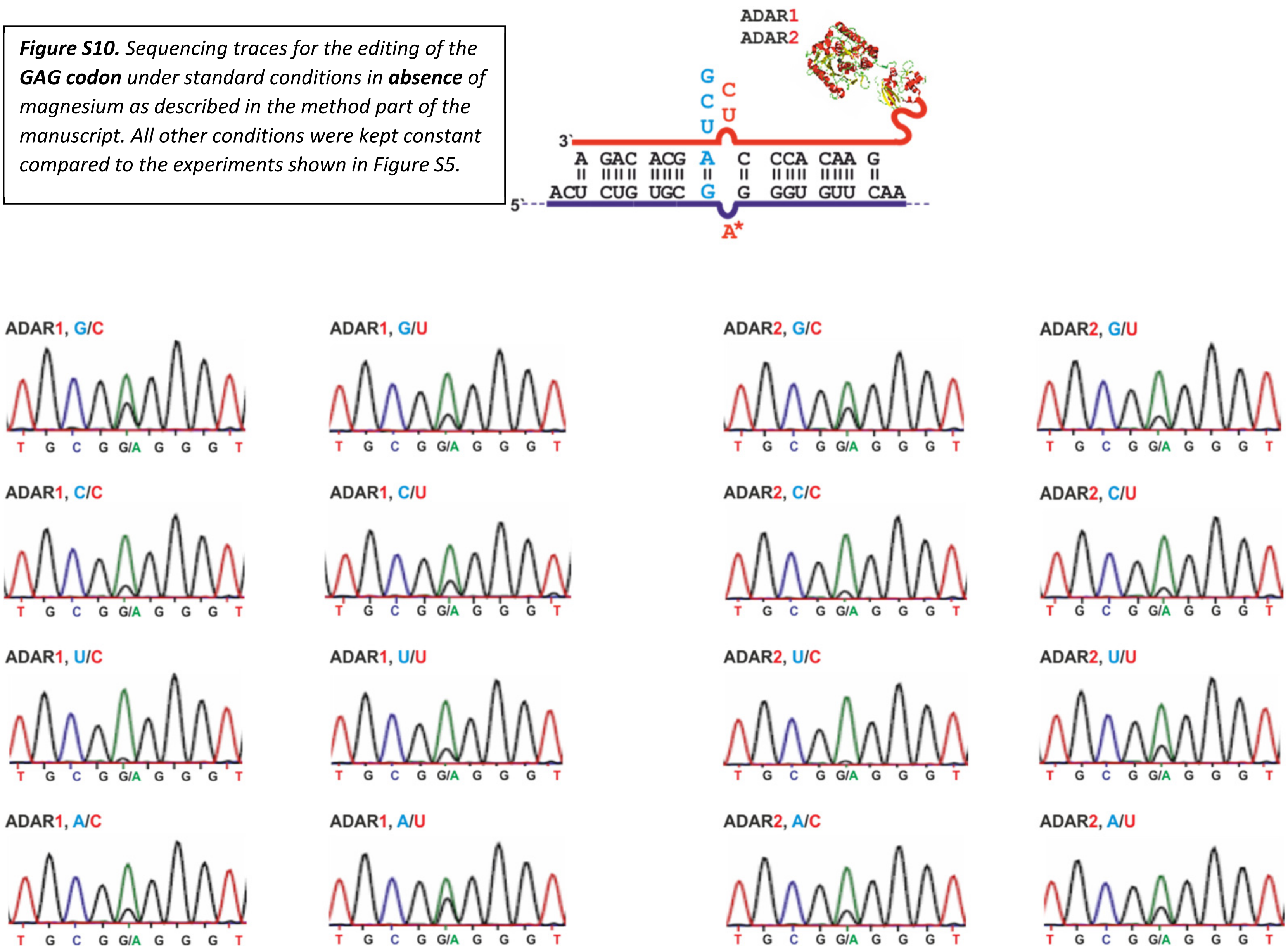

Supplement: SUPPLEMENTARY DATA [file supp_gku272_nar-03496-met-g-2013-File007.pdf]
